# Redefining the Immune System as a Social Interface for Cooperative Processes

**DOI:** 10.1371/journal.ppat.1003203

**Published:** 2013-03-21

**Authors:** Eric Muraille

**Affiliations:** Laboratoire de Parasitologie, Faculté de Médecine, Université Libre de Bruxelles, Bruxelles, Belgium; The Fox Chase Cancer Center, United States of America

## Revisiting the Red Queen Hypothesis–Derived Vision of the Immune System and Pathogens

**Figure 1:**
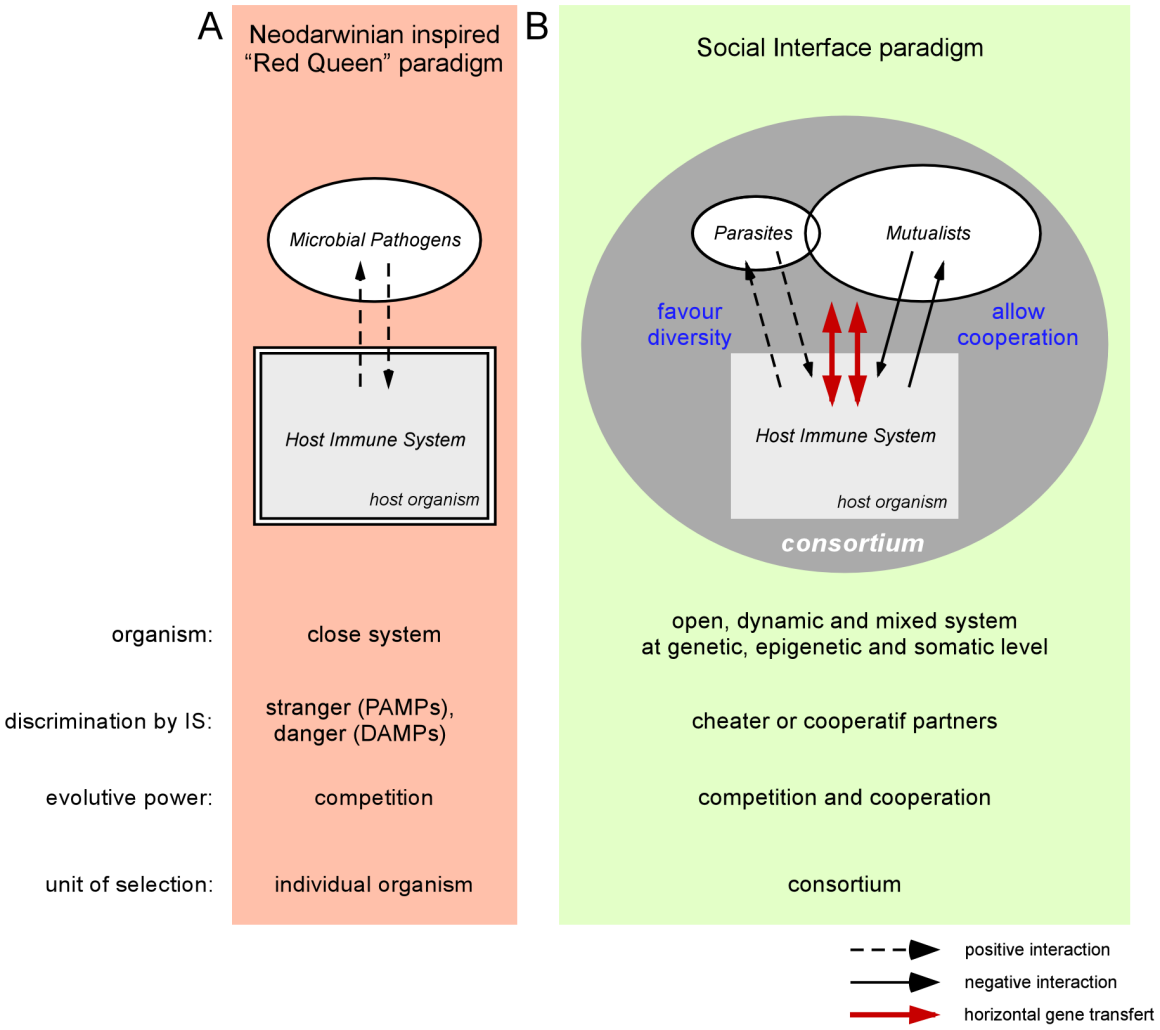
The relationship between the metazoan immune system and the microbial world. **A.** In the neo-Darwinian–inspired Red Queen paradigm, the immune system (IS) fights microbial pathogens and protects the integrity of the host organism. Pathogens are identified by the presence of PAMPs and DAMPs. **B.** In the social interface paradigm, the IS allows for the establishment of a symbiotic relationship between the host and parasites and the microbiota. Discrimination is based on a cheater or cooperative profile. In this new paradigm, the organism loses its unicity and strict boundaries and becomes an open, dynamic, and mixed consortium.

Viewed from a neo-Darwinian perspective, the main function of the metazoan immune system (IS) is to insure host integrity against invading microorganisms, which are only considered as selfish competitors that reduce the host's resources, inflict tissue damage, and ultimately compromise host fitness. Coevolution of the host and these competitors has been described as a perpetual arms race (known as the Red Queen hypothesis, Van Valen, [1]). This vision implicitly suggests that “The IS evolved under selective pressure imposed by infectious microorganisms” (Janeway, [2]) and that the ultimate objective of the IS is to conserve the integrity and sterility of the host (Figure 1A). In fact, numerous observations from microbiology and ecology have challenged this paradigm and suggest that infectious organisms and the IS play a crucial, unexpected role in evolution:

- ***The immune system performs a large list of “nonimmunological” tasks.*** Highly conserved components of the innate and adaptive IS of vertebrate are also involved in processes other than just participating in immune responses against invading microorganisms. We can take the example of phagocytosis, a well-conserved mechanism present in unicellular eukaryotes and all animal metazoans [3], that has clearly played several distinct roles during evolution. In amoebae, phagocytosis allows for the internalization of bacteria that constitute an essential source of nutriments. In metazoans, this property is mainly limited to professional phagocytes, such as macrophages, that target “altered/dying self” particles and occasionally invading microorganisms. By eliminating apoptotic cells, phagocytosis plays a major role in embryogenesis during tissue remodeling and in preventing autoimmune reactions. Similarly, complement and natural IgM [4] collaborate with phagocytic cells to eliminate apoptotic cells. Likewise, macrophages, generally considered as immune effector cells, have been shown to participate in the regulation of a growing list of processes crucial for tissue development and homeostasis, such as neuronal patterning, angiogenesis, bone morphogenesis, metabolism, and wound healing [5]. Thus, highly conserved molecules, processes, and cells of the IS can be ascribed to distinct physiological functions during evolution, with no clear link to pathogen-imposed selective pressures.
- ***The infectious organism promotes host genetic diversity.*** Genetic variation in natural populations is a prime prerequisite for the response of populations to selection pressure. In antagonist coevolution, hosts are selected to evade infection whereas the pathogen is selected to infect the host. In 1949, Haldane proposed that an important positive impact of this phenomenon is the maintenance of high genetic diversity among both host and pathogen populations: “Just because of its rarity it will be resistant to diseases which attack the majority of its fellows.” This hypothesis has since been largely confirmed [6], [7]. A recent study even suggests that pathogens have a higher impact on human genetic diversity than climate conditions [8].
- ***Infection favors free circulation of genetic innovations.*** The sequencing of whole genomes has demonstrated that symbiotic microorganism interactions favor horizontal genetic transfers (HGT) and thus the rapid spread in many lineages of genetic innovations that would have otherwise taken millions of years [9]. A fascinating example demonstrating that infection can contribute to biological innovation is the acquisition by host vertebrates of recombinase-activating genes (RAGs) [10] and Syncitin [11] genes from viruses. These viral genes have allowed for development of the adaptive IS and the syncytiotrophoblast, respectively. Thus, infectious organisms appear to have been essential during evolution to maintain diversity and allow free circulation of genes by HGT, transforming the “tree of life” proposed by neo-Darwinian theories into a dynamic and interconnected “net of life.”
- ***Chronic infection can improve host resistance to infection*** **.** Infectious organisms are in constant competition with other infectious organisms to invade and persist in their specific host (a form of “apparent competition”). They can compete together by cross-reacting with the host immune response and thus enhance the general resistance of the host to infection. In arthropods [12] and mammals [13], several studies have shown that latent infections by viral or bacterial pathogens are beneficial to the host's health by protecting it against other infectious organisms.
- ***Specific immunity against pathogens can affect the result of intraspecies or near-species competition and thus the evolution of species.*** After the peak of an epidemic infection, a population generally contains an enhanced frequency of resistant individuals and the pathogen is frequently conserved in healthy carriers. When a protected (colonized) population encounters a naive population (uncolonized), the immune status of experienced individuals constitutes a potent competitive weapon against nonimmune individuals. This phenomenon is well-described in prokaryotes [14], mammals [15], and during human history [16].
- ***Chronic infection by pathogens can play an essential role in the host life cycle.*** Well-documented examples of this phenomenon are the interdependence of Wolbachia [17] or polydnaviruses [18] with their arthropod hosts.
- ***Cooperative symbiosis plays a major role in evolution.*** Neo-Darwinian–inspired paradigms of evolution are mainly based on competition between organisms. However, a growing body of data supports the idea that cooperative behavior is present at every level within living kingdoms. Bacteria often self-assemble in complex structures such as mono- or multispecies biofilms that stand up to environmental threats better [19]. Eukaryotic cells derive from symbiotic events between ancestral eukaryotic cells, prokaryotes, and possibly DNA viruses [20]. Numerous unicellular organisms, such as *Myxocossus xanthus* [21], *Saccharomyces cerevisiae* [22], or *Dictyostelium discoideum* amoebae [23], can be found in a pluricellular state formed by aggregation. This structure can be mobile (slug), allowing for better resistance to predation and migration to new territories, or fixed (fruiting body), allowing for the production and dissemination of spores. These examples illustrate how prokaryotic and eukaryotic unicellular organisms can build consortia that promote cell differentiation and task specialization. As such, metazoans can be viewed as stabilized consortia of social unicellular eukaryotes in association with a complex viral, bacterial, and fungal flora. This flora, termed the microbiota, is essential to the metabolism of various nutrients, the development and regulation of the IS, and the fight against infection by competition [24]–[26]. Alteration of microbiota frequently opens the door to opportunist infections and to obesity and inflammatory disease such as type 2 diabetes. The influence of microbiota could be even more important than previously anticipated, as recent observations suggest that commensal gut bacteria can influence mating preference and thus sexual selection in *Drosophila melanogaster* [27], while a *Lactobacillus* strain appears to affect emotional behavior in mice [28]. The interactions between microbiota and the host IS are highly dynamic. Studies in germ-free mice have demonstrated the importance of microbiota in the full maturation of the IS [24]. In turn, the establishment of a cooperative microbiota-host equilibrium within the digestive tract requires a fully competent IS [29]. Finally, at a higher level, insect and human societies are well-described examples of highly cooperative structures. Thus, we can conclude, like Strassmann and Queller [30], that all organisms are cooperative social groups, or consortia, frequently displaying interspecies cooperation. As the composition of these consortia is dynamic and transmitted vertically to the next generation, they display partial Lamarckian properties, as discussed in detail by Rosenberg [31].

## The Social Interface Hypothesis

Simple observations of ecosystems lead us to conclude that mutually beneficial interactions (cooperative behavior) are prevalent throughout the biological world, both within and across species and at all levels from genes to societies. As suggested by Maynard Smith [32], cooperative interactions seem to be the key to understanding the major transition in life. However, their selection and relevance have challenged theorists for decades, in part because of the “Prisoner's Dilemma” [33], which has dominated the literature on cooperation. This paradigm depicts how two unrelated players benefit from mutual cooperation, and how a cheating player can increase its advantage by reaping the benefits of the cooperating population without contributing to public goods (PGs). Eventually, the entire population may collapse when the proportion of cheaters increases beyond a critical point, a scenario known as “the tragedy of the commons,” which was initially described by Hardin [34]. Explaining how selection can promote and stabilize a trait that benefits another individual constitutes one of the greatest challenges in evolutionary biology.

Limitation of the distribution of PGs to kin organisms was the first solution originally suggested by Hamilton in the context of animal social behavior [35]. “Kin selection” could explain the selective pressure leading to close and permanent aggregation of genetically related cells in clonal pluricellular structures evolving toward actual metazoans and several traits of metazoan societies. However, this solution does not explain, for example, the maintenance of cooperation in multispecies biofilms and the establishment of cooperative symbiotic relationships between metazoans and their microbiota. A second condition to stabilization of cooperation has been identified [30], [36]. During evolution, the benefits of consortium formation necessarily promote the selection of policing mechanisms that are crucial to reducing conflicts inside the consortium. If competition is banished, each member of a consortium could increase its own success only by increasing the efficiency and productivity of the whole group. Thus, repression of competition within consortia joins kin discrimination as the second major force in the history of life that has shaped the evolution of cooperation. In practical, these “policing mechanisms” must be able to discriminate between cooperative partners and cheaters in order to neutralize or contain cheaters. Policing mechanisms can be identified in all consortia:

- Prokaryotes are confronted with the constant threat of phage predation and, consequently, have developed a wide variety of mechanisms against them. Four major systems have been described [37]: RM (restriction modification), DGR (diversity-generating retroelement), abi (abortive infection), and CRISPR (clustered regularly interspersed palindromic repeats). This last system can even confer adaptive immunity to viruses [38] and allow for the long-term memorization of viral cheaters. In addition, biofilms display various systems to limit the appropriation of PGs by selfish bacteria [39].
- When social unicellular organisms, such as the *D. discoideum* amoeba, form a multicellular structure, there is no guarantee that all amoebae are genetically identical and thus the aggregate is frequently chimeric with incomplete relatedness. As a fraction of cells differentiate into viable spores while others “self-sacrifice” to give rise to the stalk, the control of differentiation is critical to ensure that cheaters do not exploit the cooperative process to their sole benefit. As observed in biofilms, aggregates display several anticheating mechanisms such as kin discrimination by an adhesion system, lottery-like role assignment, pleiotropy, noble resistance [40], and differentiated patrolling phagocytic sentinel cells [41].
- Metazoans possess a complex IS displaying a large panel of effector mechanisms able to detect and fight all types of invading pathogenic organisms but also syngeneic cheater/selfish (tumor) cells. Cheaters could be identified by the innate IS that has evolved detection mechanisms to react to patterns of pathogenesis (POPs) [42]. POPs can be defined as a combination of signals including the production of microbial-associated molecular patterns (MAMPs) and damage-associated molecular patterns (DAMPs) in specific conditions of infection. Adaptive IS allows for the long-term memorization of cheaters and even the partial transmission of past experience to descendants by the maternal transfer of antibodies to newborns.
- The extraordinary ecological success of social insects has been attributed to their ability to achieve high cooperation and to cope with the rich infectious microbial community inhabiting their nests. This has required the development of a complex “social IS” [43], [44]. Reproductive conflicts in insect societies are inevitable because they are almost always related families, not clones. Social IS implicates “worker policing” that resolves conflicts by coercion and constraints [44]. In some cases, these tasks are even performed by specialized workers [45]. Social IS also involves the communication of information about the presence of infectious organisms, mutual grooming scaled to their presence, and removal of diseased individuals from the nest [43].

It is important to observe that partner tolerance, identification of syngeneic cheaters (selfish mutant, cancer cells) and allogeneic cheaters (infectious organisms), as well as mechanisms to exclude and memorize them, which are all usually considered as characteristics of the vertebrate IS, exist in all consortia. I propose to give a general name to these diverse structures that allow for dialogue between partners and the formation of stable consortia at each level of life complexity: the “social interface” (SI). The SI can take various forms: primitive exclusion mechanisms among social unicellular organisms, complex metazoan IS, or central nervous systems that have allowed for the development of social IS among metazoan societies. SI are indispensable to the stabilization of a consortium by kin discrimination and reduction of conflict.

## Cost of the Social Interface and Biological Identity

There many costs and consequences of acquisition of SIs for all organisms. Obvious costs of the SI are those for energy and collateral damage due to policing mechanisms that neutralize cheaters such as the inflammatory immune response [46]. However, another important cost is the risk of autoimmunity. Autoimmunity, classically defined in mammalian IS, is the failure of the IS to recognize what is self and what is foreign, resulting in an immune response against self. I propose that autoimmunity is a risk associated with all SIs and thus shared by all consortia. In keeping with this argument, potential sources of the autoimmune reaction have been reported in bacteria and linked to the CRISPR system [47] and in social amoebae [48].

The SI paradigm also leads us to reconsider the role of the IS in the determination of the “biological identity” of organisms. The nature of this biological identity was debated in the nineteenth and twentieth centuries by a great number of authors [30], [49]. What is self is a fundamental question in biology. Since Burnet [49], the ability to discriminate between self and nonself has been associated with the metazoan IS. However, as previously mentioned, this discriminating property has been reported in social bacteria [47] and amoebae [48] and can be considered as a general consequence of the acquisition of an SI. Based on the unique ability to discriminate between cooperative and cheater partners of an SI, the self becomes the sum of cooperative and interdependent partners. At the genetic, epigenetic, and somatic levels, this is necessarily an open, dynamic, and mixed system (Figure 1B).

## The Metazoan Immune System as an Organ Devoted to Symbiosis

**Figure 2:**
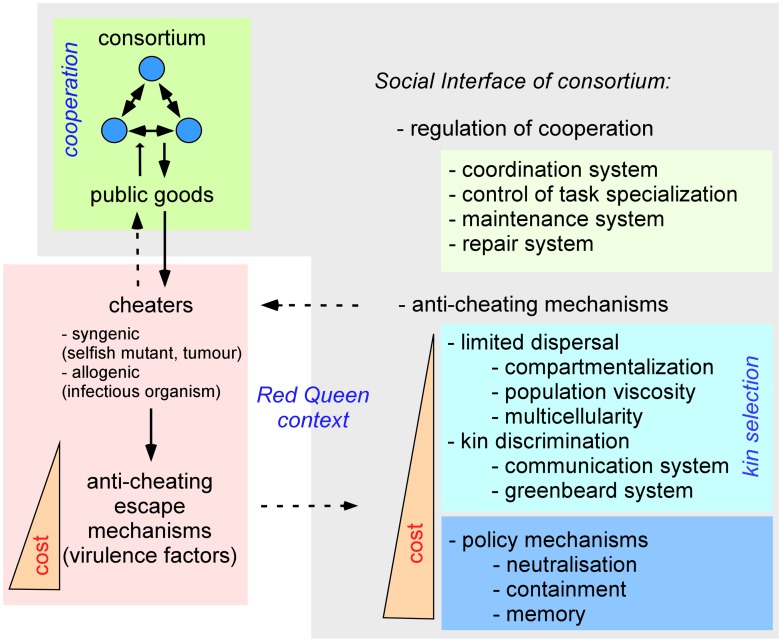
Role of the social interface in consortia. The social interface (SI) appears to be responsible for management of the cooperation of syngeneic cells in consortia, detection of selfish/cheater behavior of syngeneic or allogeneic cells, and control and memorization of cheaters. To escape policing mechanisms of SIs, cheaters develop anticheating escape mechanisms, leading to “Red Queen” antagonistic coevolution.

In conclusion, I propose that the innate and adaptive metazoan IS has evolved under selective pressure favoring symbiosis, a source of genetic diversity, HGT, and cooperation that globally promote better adaptation to selective pressure. In this view (Figure 2), the metazoan IS appears, like all SIs, to be responsible for: (i) management of the cooperation of syngeneic cells, which may explain the numerous functions of the IS in the development and maintenance of organisms in the absence of infection; (ii) detection of selfish/cheater behavior of syngeneic or allogeneic cells; and (iii) elimination and memorization of cheaters. This invites reinterpretation of the condition for IS activation. The “danger signal” proposed by Matzinger [50] and POPs [42] could be redefined as “selfish/cheater signals.” The importance of selfish/cheater behavior in activation of the IS is demonstrated by the ability of the IS to tolerate our allogeneic cooperative microbiota and fight syngeneic selfish cells (tumors).

The crucial importance of infections for the evolution/adaptation of life and maintenance of the fitness of consortia strongly suggests that complete neutralization of infection by immune systems cannot be favorable to host adaptation. Consequently, I propose that all SI (including the vertebrate IS) must partially tolerate selfish/cheater/infectious organisms. Only cheaters strongly affecting the fitness of consortia must be eliminated. This could explain the fact that all organisms are, in natural conditions, always infected. These infections are not the consequence of failure of the immune system but a logical consequence of the necessity to partially tolerate infectious organisms, a source of HGT, new potential cooperative partners, etc. This is in total opposition to the classical view of the IS, considered only as a defense mechanism conferring “sanctuary status” on the organisms and obsessed with the eradication of infection and host sterility.
